# High Proportions of Multidrug-Resistant *Acinetobacter* spp. Isolates in a District in Western India: A Four-Year Antibiotic Susceptibility Study of Clinical Isolates

**DOI:** 10.3390/ijerph15010153

**Published:** 2018-01-19

**Authors:** Ingvild Odsbu, Smita Khedkar, Uday Khedkar, Sandeep S. Nerkar, Ashok J. Tamhankar, Cecilia Stålsby Lundborg

**Affiliations:** 1 Department of Public Health Sciences, Karolinska Institutet, 17177 Stockholm, Sweden; san.ner1978@gmail.com (S.S.N.); ejetee@gmail.com (A.J.T.); cecilia.stalsby.lundborg@ki.se (C.S.L.); 2 Bac-Test Laboratory, College Road, Nashik 422005, Maharashtra, India; mail@bactestlab.com (S.K.); udayk_nsk@sancharnet.in (U.K.); 3 Indian Initiative for Management of Antibiotic Resistance, Department of Environmental Medicine, R.D. Gardi Medical College, Ujjain 456006, India

**Keywords:** *Acinetobacter*, India, multidrug resistance, antibiotic resistance, antibacterial susceptibility testing

## Abstract

The purpose of the study was to determine the proportions of multidrug-resistant (MDR) *Acinetobacter* spp. isolates from the district of Nashik in Western India during the period from 2011–2014. Antibacterial susceptibility testing of isolates from inpatients and outpatients was performed using Kirby–Bauer disc diffusion method to determine inhibitory zone diameters. Proportions of non-susceptible isolates were calculated from the antibacterial susceptibility data. MDR was defined as an isolate being non-susceptible to at least one antibacterial agent in at least three antibacterial categories. The change in proportions of MDR isolates; extended-spectrum β-lactamase (ESBL)-producing isolates; and non-susceptible isolates to specific antibacterial categories over calendar time was investigated by logistic regression. The proportions of MDR and ESBL-producing isolates ranged from 89.4% to 95.9% and from 87.9% to 94.0%; respectively. The proportions of non-susceptible isolates to aminoglycosides; carbapenems; antipseudomonal penicillins/β-lactamase inhibitors; cephalosporins; folate pathway inhibitors; or penicillins/β-lactamase inhibitors exceeded 77.5%. Proportions of fluoroquinolone and tetracycline non-susceptible isolates ranged from 65.3% to 83.3% and from 71.3% to 75.9%; respectively. No changes in trends were observed over time; except for a decreasing trend in fluoroquinolone non-susceptible isolates (OR = 0.75 (95% CI, 0.62–0.91)). Significantly higher proportions of non-susceptible; MDR and ESBL-producing isolates were found among isolates from the respiratory system compared to isolates from all other specimen types (*p* < 0.05). High proportions of MDR *Acinetobacter* spp. isolates were observed in the period from 2011–2014. Antimicrobial stewardship programmes are needed to prevent the emergence and spread of antibiotic resistance.

## 1. Introduction

*Acinetobacter* species (spp.) are Gram-negative coccobacilli that cause serious healthcare-associated infections like ventilator-associated pneumoniae, blood stream infections, urinary tract infections, and wound infections in critically ill patients [1,2]. Community-acquired infections like skin and soft tissue infections in relation to trauma are also caused by the genus *Acinetobacter* [1]. The bacterium *Acinetobacter baumannii* is the most clinically important of the bacteria belonging to this genus [3]. During the last three decades *A. baumannii* isolates have become resistant to more and more classes of antibiotics due to both intrinsic and acquired resistance mechanisms [2,4]. For a long time carbapenems was the most reliable treatment option for infections caused by *Acinetobacter* spp., but carbapenemase-producing isolates are emerging globally [2,5]. Alternative treatment options are the last-resort drugs polymyxins (colistin or polymyxin B) and tigecycline, or a combination of one of these classes with a second agent [2,6,7]. *Acinetobacter* spp. isolates that are resistant to colistin or polymyxin B have been reported worldwide [8,9]. Due to the rising levels of resistant isolates therapeutic options are limited or even absent in some cases of infections with pandrug-resistant bacteria. *Acinetobacter* spp. are adapted to persistence in hospital settings and are difficult to eradicate [2]. Infections caused by *Acinetobacter* spp. are associated with increased mortality [10,11], increased length of hospital stay [11], and higher costs [12]. The high prevalence of resistant isolates towards many classes of antibiotics makes initiation of effective empiric treatment challenging [13]. It is of great importance with local and national surveillance data on the prevalence of resistant *Acinetobacter* spp. isolates, both to inform local treatment guidelines and to detect outbreaks and changes in trends. Both regional and national surveillance systems are lacking in India. Hence, there is great need for long-term studies on the proportions and trends in antibiotic resistance among *Acinetobacter* spp. isolates to gain more knowledge about the development and spread of antibiotic resistance in India. In this study we have examined the proportions of and trends in multidrug-resistance (MDR) among *Acinetobacter* spp. isolates from the district of Nashik in Western India during the period 2011–2014. 

## 2. Materials and Methods

### 2.1. Study Setting

Antibacterial susceptibility testing of clinical *Acinetobacter* spp. isolates were performed at the Bac-Test Laboratory, an ISO certified laboratory that serves various hospitals, clinics and diagnostic laboratories in the district of Nashik [14]. The district belongs to the state of Maharashtra in the western part of India and has a population of more than 6.1 million and covers an area of 15,582 km^2^ [15]. 

### 2.2. Study Material

The data were collected in the period from 1 January 2011 to 31 December 2014. Information about type of patient (in- or outpatient), type of specimen (blood, respiratory etc.), date of specimen collection, and the results of the antibacterial susceptibility testing for every isolate were recorded in the WHONET software [16]. The data were collected at the genus level and not the species level. The data material consisted of a total of 763 *Acinetobacter* spp. isolates. Eighteen isolates were excluded due to lack of information about patient type, and another four isolates were excluded since they were tested for less than three antibacterial categories (see definition of MDR in the Section 2.4. Statistical analyses). Hence, the final data set consisted of 741 *Acinetobacter* spp. isolates. 

The data material used in this study was retrospective data from routine analysis of antibacterial susceptibilities. The data were anonymous since no patient identifiers were available, and it would therefore not be possible to obtain informed consent from the patients.

### 2.3. Laboratory Methods

Antibiotic susceptibility testing was performed using the Kirby–Bauer disk diffusion method (with disks from HiMedia Laboratories, Mumbai, India and Becton, Dickinson, Franklin Lakes, NJ, USA) according to the most updated Clinical and Laboratory Standards Institute (CLSI) guideline at the time of analysis [17,18,19]. The double disk synergy test was used to screen for extended-spectrum β-lactamase (ESBL)-producing isolates [17]. 

### 2.4. Statistical Analyses

The recorded inhibitory zone diameters were interpreted according to the clinical zone diameter breakpoints provided by the CLSI guideline used at the time of the antibacterial susceptibility testing [17]. Susceptible bacterial isolates were classified as “susceptible”, whereas intermediate susceptible and resistant bacterial strains were classified as “non-susceptible”. Multidrug resistance (MDR) was defined using the criteria proposed by the European Centre for Disease Prevention and Control (ECDC) and the Centers for Disease Control and Prevention (CDC) [20]. According to the criteria and available data in the data set, an *Acinetobacter* spp. isolate was classified as MDR if the isolate was non-susceptible to at least one antibacterial agent in at least three of the following antibacterial categories; aminoglycosides (gentamicin, tobramycin, or amikacin), carbapenems (imipenem, meropenem, or doripenem), fluoroquinolones (ciprofloxacin or levofloxacin), antipseudomonal penicillins/β-lactamase inhibitors (piperacillin-tazobactam or ticarcillin-clavulanic acid), cephalosporins (cefotaxime, ceftriaxone, ceftazidime, or cefepime), folate pathway inhibitors (trimethoprim-sulphamethoxazole), penicillins/β-lactamase inhibitors (ampicillin-sulbactam), and tetracyclines (tetracycline or doxycycline). Polymyxins (colistin or polymyxin B) were not included in the analyses due to lack of clinical breakpoints in the CLSI guideline [17]. Non-susceptibility to an antibacterial category was defined as being non-susceptible to at least one antibacterial agent in an antibacterial category [20]. Changes in clinical zone diameter breakpoints for meropenem and imipenem in 2014 were taken into account when classifying the isolates as “susceptible” or “non-susceptible”. No clinical zone diameter breakpoints were available for doripenem before 2014. For the isolates tested for susceptibility to doripenem in the period from 2011–2013, the clinical zone diameter breakpoints for meropenem and imipenem in the same period were used.

Proportions of non-susceptible isolates (number of non-susceptible isolates as a proportion of total isolates tested) were calculated from the antibacterial susceptibility data. Calculated proportions of non-susceptible isolates were compared between inpatients and outpatients, and between various specimen types using Chi square tests. A two-sided *p*-value < 0.05 was considered statistically significant. Odds ratios (ORs) of non-susceptible isolates associated with 1-year increment in the period 2011–2014 were estimated along with 95% confidence intervals using logistic regression.

Statistical analyses were performed using Stata version 14 (Stata Corp., College Station, TX, USA).

## 3. Results

### 3.1. Acinetobacter spp. Isolates

Out of the 741 *Acinetobacter* spp. isolates included in the study, the majority of the isolates were obtained from the respiratory system (50.7%; includes samples from bronchoalveolar lavage, sputum, throat and trachea), followed by pus (18.3%) and blood (15.4%) samples. Inpatients and outpatients contributed to 88.5% and 11.5% of the samples, respectively. 

### 3.2. High Proportions of MDR Acinetobacter spp. Isolates during the Period from 2011–2014

**Figure 1:**
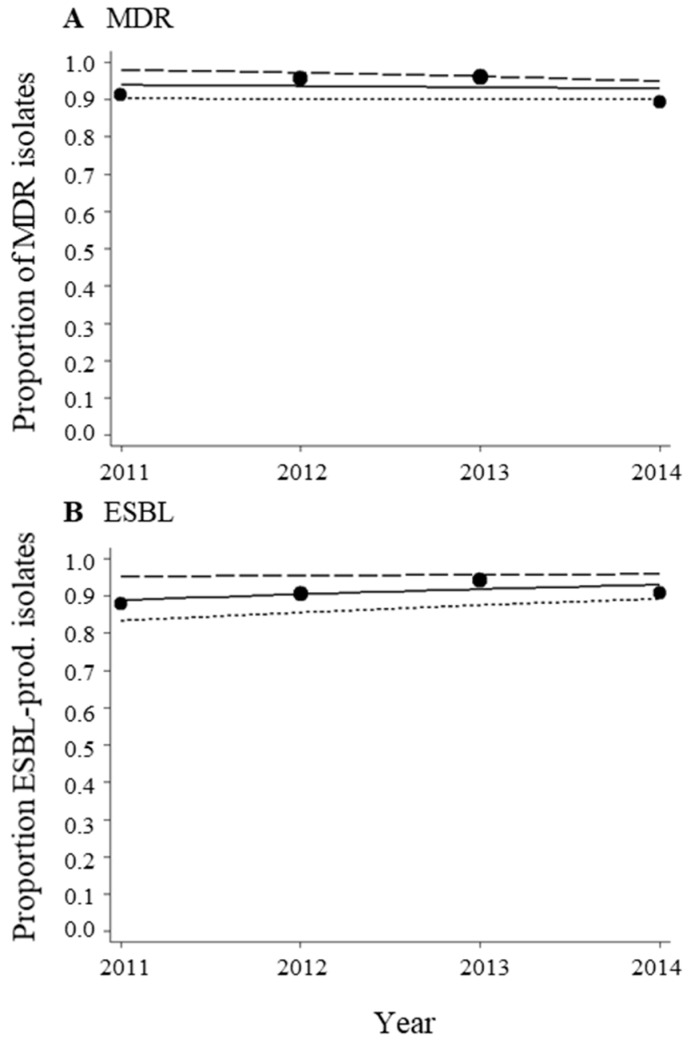
Proportions of multidrug-resistant (MDR) (**A**) and extended-spectrum β-lactamase (ESBL)-producing (**B**) *Acinetobacter* spp. isolates. The data were fitted with a logistic regression model (solid line = all isolates, long dashes = isolates from respiratory samples, short dashes = isolates from all other specimen types). Each bubble shows the proportion of isolates per year in the period from 2011–2014. The size of the bubbles represents the sample size (see Table A1). The total numbers of isolates tested for MDR and ESBL were 741 and 690, respectively.

**Figure 2:**
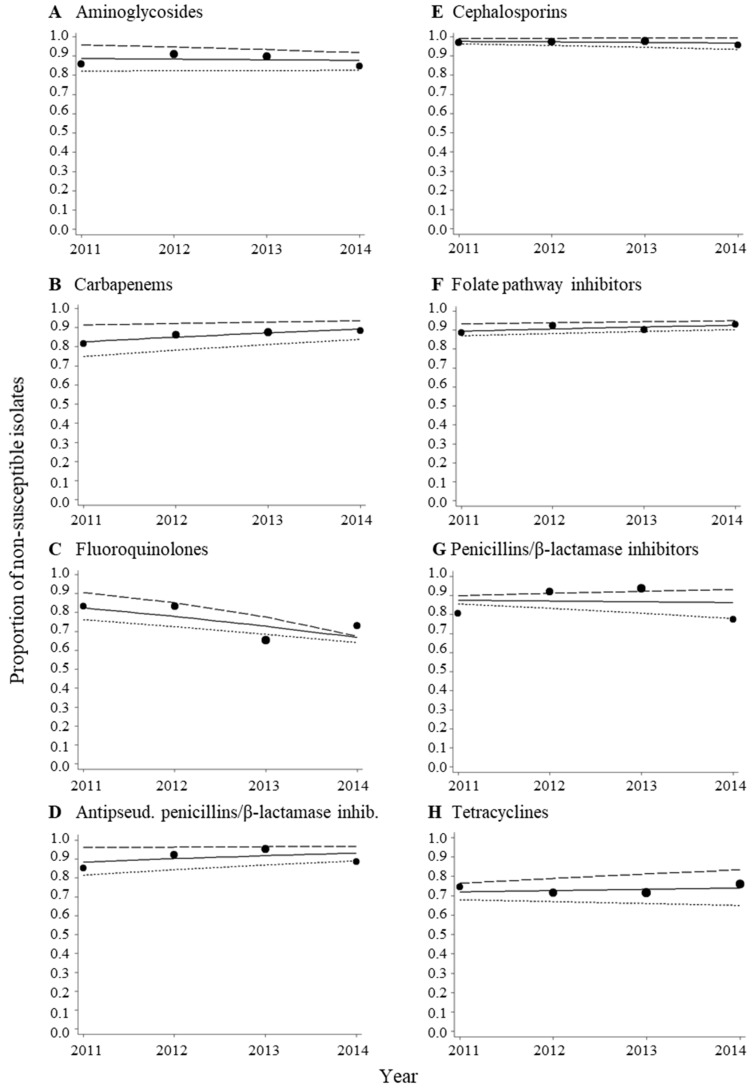
Proportions of non-susceptible *Acinetobacter* spp. isolates according to antibacterial category and year. The antibacterial categories were aminoglycosides (**A**, *n* = 716), carbapenems (**B**, *n* = 732), fluoroquinolones (**C**, *n* = 578), antipseudomonal penicillins/β-lactamase inhibitors (**D**, *n* = 710), cephalosporins (**E**, *n* = 722), folate pathway inhibitors (**F**, *n* = 456), penicillins/β-lactamase inhibitors (**G**, *n* = 675), and tetracyclines (**H**, *n* = 405). The data were fitted with a logistic regression model (solid line = all isolates, long dashes = isolates from respiratory samples, short dashes = isolates from all other specimen types). Each bubble shows the proportion of isolates per year. The size of the bubbles represents the sample size (see Table A1). *n* = total number of isolates in the period 2011–2014.

**Figure A1:**
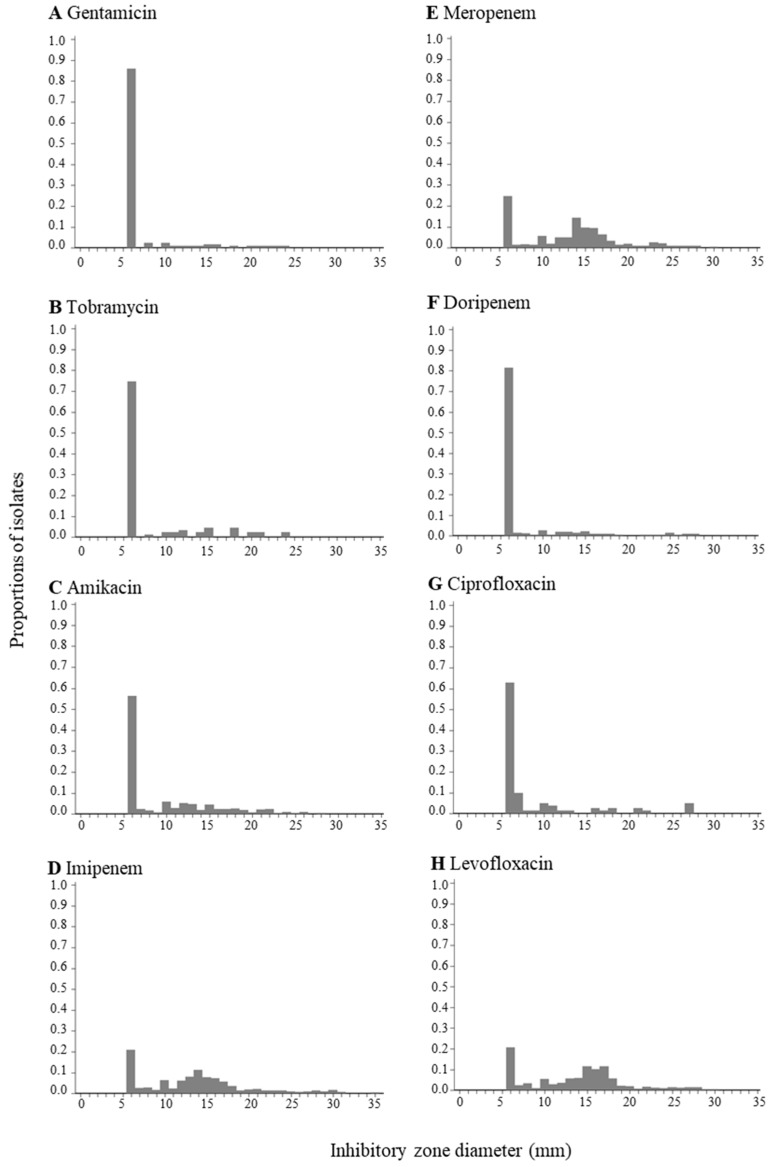

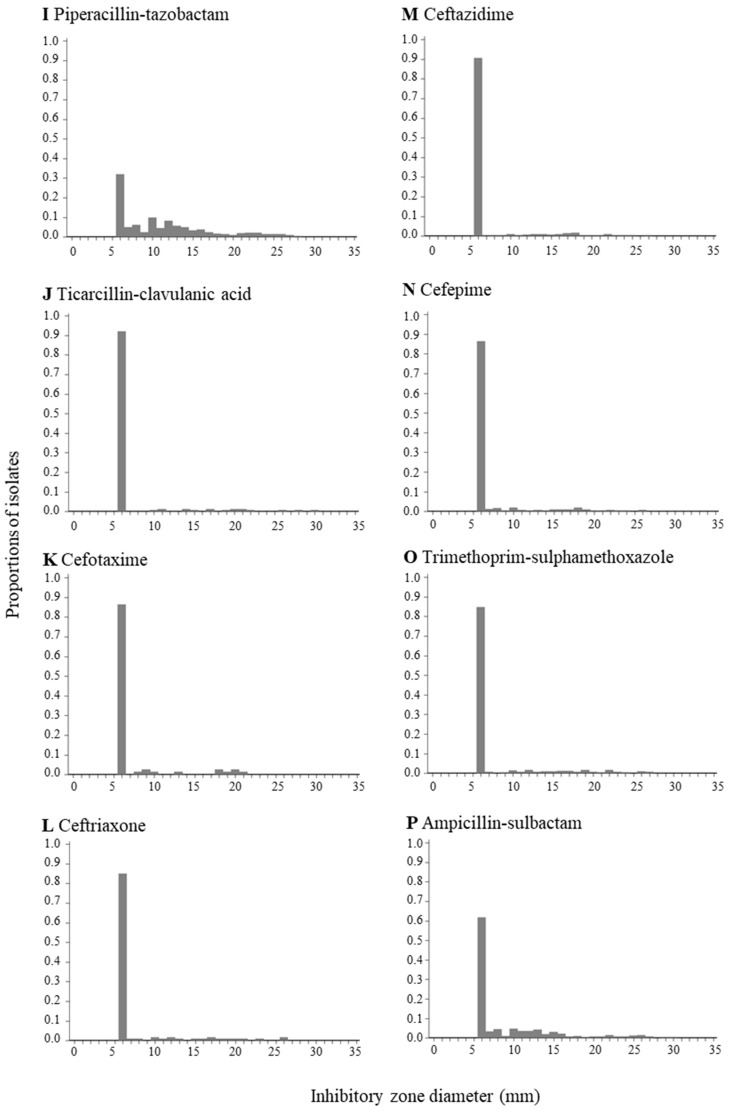

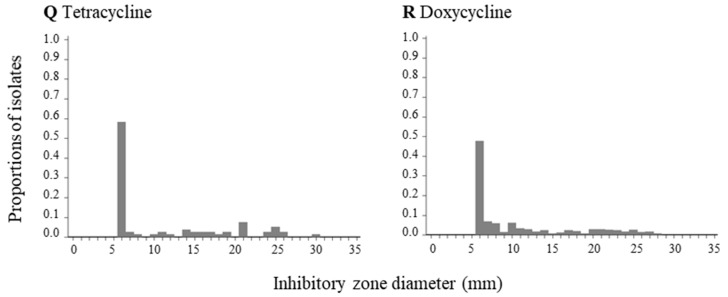
Distribution of inhibitory zone diameters (mm) according to antibacterial agent (**A**–**R**) in the period 2011–2014.

The proportions of MDR isolates ranged from 89.4% to 95.9% in the study period (Figure 1A and Table A1). For isolates that tested positive for ESBL, the proportions ranged from 87.9% to 94.0% (Figure 1B and Table A1). High proportions of non-susceptible isolates were found for the antibacterial categories aminoglycosides (84.7–91.0%), carbapenems (81.6–88.3%), antipseudomonal penicillins/β-lactamase inhibitors (85.1–95.3%), folate pathway inhibitors (88.7–93.1%), and penicillins/β-lactamase inhibitors (77.5–93.7%) (Figure 2 and Table A1). For cephalosporins, the proportions of non-susceptible isolates were found to be even higher, ranging from 95.7% to 97.7% (Figure 2E and Table A1). The proportions of non-susceptible isolates to fluoroquinolones and tetracyclines were found to range from 65.3% to 83.3% and from 71.3% to 75.9%, respectively (Figure 2C,H, and Table A1). The distributions of the inhibitory zone diameters for the antibacterial agents are shown in Figure A1.

When comparing the proportions of MDR and ESBL-producing isolates between inpatients and outpatients, no statistically significant differences were found (Table 1). A higher proportion of non-susceptible isolates to aminoglycosides (*p* = 0.008) and antipseudomonal penicillins/β-lactamase inhibitors (*p* = 0.048) were found in inpatients. For the other antibacterial categories, no statistically significant differences between inpatients and outpatients were found (Table 1). Significantly higher proportions of non-susceptible, MDR and ESBL-producing isolates were found among isolates from the respiratory system compared to isolates from all other specimen types (*p* < 0.05, Table 2).

### 3.3. No Change in Trends of MDR Acinetobacter spp. Isolates during the Period from 2011–2014

An analysis of trends using logistic regression revealed that there were no changes in trends for MDR and ESBL-producing isolates (Table 3). No changes in trends were also observed for non-susceptibility to all antibacterial categories except for fluoroquinolones. Per every year increment in the period from 2011–2014 there was a 25% decrease in the probability of an *Acinetobacter* spp. isolate being non-susceptible to fluoroquinolones (OR = 0.75 (95% CI, 0.62–0.91)) (Table 3). When stratifying on type of specimen (respiratory samples versus samples from other specimen types), there was a 40% decrease in the probability of an isolate being non-susceptible to fluoroquinolones for the respiratory samples (OR = 0.60 (95% CI, 0.43–0.84)) (Figure 2C). No change in trend for fluoroquinolone non-susceptibility was observed for the samples from other specimen types (OR = 0.82 (95% CI, 0.65–1.04) (Figure 2C). In the period from 2011–2014, there was a higher proportion of respiratory samples, as well as a slightly higher proportion of samples from inpatients, collected towards the end of the period compared to samples from other specimen types and outpatients (Table A2). 

For MDR and ESBL-producing isolates, as well as all other antibacterial categories, no differences in trends were observed for respiratory samples compared to samples from other specimen types (Figure 1 and Figure 2). 

## 4. Discussion

In this study we have analyzed data on antibacterial susceptibility over a four years period among *Acinetobacter* spp. isolates from healthcare-associated- and community-acquired infections in the district of Nashik in Western India. We found high proportions of MDR and ESBL-producing isolates, as well as high proportions of non-susceptible isolates to aminoglycosides, carbapenems, fluoroquinolones, antipseudomonal penicillins/β-lactamase inhibitors, cephalosporins, folate pathway inhibitors, penicillins/β-lactamase inhibitors, and tetracyclines.

No changes in non-susceptibility trends were observed in the period from 2011–2014, except for fluoroquinolone non-susceptible isolates where we found a decreasing trend over time. The decreasing trend in fluoroquinolone non-susceptible isolates could possibly be explained by a change in type of sample collected over time, particularly a reduction in the proportions of respiratory samples for which we found significantly higher proportions of fluoroquinolone non-susceptible isolates (Table 2). However, in the period from 2011–2014, the proportions of respiratory samples tested for fluoroquinolone non-susceptibility rather increased (Table A2). When stratifying on type of specimen, we found a decreasing trend among isolates from the respiratory samples that explains the decreasing trend observed for all isolates tested for fluoroquinolone non-susceptibility. Since a decreasing trend was observed for the respiratory samples despite an increased sampling of the specimen type, the finding might reflect a true drop in fluoroquinolone non-susceptible isolates among respiratory samples. An explanation for the decreasing trend observed could be due to decreasing use of fluoroquinolones to treat respiratory infections. We did not have data on antibiotic consumption, neither from the individual patient nor at the aggregated level from the district of Nashik, so this will only be a speculation. 

More surveillance data over a longer time period are needed to be able to conclude whether there is a steady decrease in the proportion of fluoroquinolone non-susceptible isolates or whether there will be an increase in the proportions of fluoroquinolone non-susceptible isolates in the coming years. Fluctuating proportions of resistant *Acinetobacter* spp. isolates over time has been observed in other long-term studies [21,22,23,24,25]. In another study on antibacterial susceptibility over an 11 years period among *E. coli* and *Klebsiella* spp. isolates from the district of Nashik we also found fluctuating trends in resistance over time (unpublished results). The fluctuating trends might reflect differences in the annual burden of infections or changes over time in the choice of empiric drugs for treatment of infections. The decision on which antibiotic to choose can also be influenced by external factors like drug price, drug availability and drug promotion practices. Information about these factors is difficult to obtain and were not available in our study. 

The majority of the *Acinetobacter* spp. isolates were obtained from respiratory samples. This finding is consistent with the distribution of specimen types among *Acinetobacter* spp. isolates found in other studies [26,27,28,29,30,31,32]. The high prevalence of respiratory samples in our data indicates that *Acinetobacter* spp. most frequently cause respiratory disease in this setting. We found significantly higher proportions of MDR, ESBL-producing and non-susceptible isolates among isolates from the respiratory system compared to isolates from other specimen types. One explanation for the higher prevalence of MDR isolates from respiratory samples could be that the *Acinetobacter* strains causing ventilator-associated pneumonia or other healthcare-associated pneumoniae are more frequently exposed to antibiotics compared to strains causing other types of infection. Although we found significantly higher proportions of MDR isolates from respiratory samples, the proportions of MDR isolates from other specimen types were still high, which indicates that empiric treatment of all types of infections caused by *Acinetobacter* spp. is difficult.

*Acinetobacter* spp. have a natural MDR phenotype [33]. High proportions of MDR and ESBL-producing isolates were found in our study, and increasing and high proportions of MDR *Acinetobacter* spp. isolates have also been reported globally [24,34,35]. Of particular concern is the high proportion of carbapenem non-susceptible isolates observed throughout the study period. Another study from a tertiary care center in Pune, also in Western India, reported 42% carbapenem-resistant *Acinetobacter* spp. isolates from patients admitted to the intensive care unit in the period 2011–2013 [28]. Carbapenem resistance rates in *Acinetobacter* spp. isolates from other regions of India have been reported to be from 67% to 74% in the period 2009–2013 [21], from 0% to 84% in the period 2002–2008 [36], and 32% in the period 2010–2011 [25]. As opposed to our study, these studies are single-site studies. Our data material cover clinical *Acinetobacter* spp. isolates from a large number of clinics and hospitals in the district of Nashik, and reflect the resistance proportions in a larger region. The high proportions of carbapenem non-susceptible isolates in the district of Nashik indicate that carbapenems often will be inappropriate as empiric therapy to treat infections caused by *Acinetobacter* spp. in this region. A recent study from the U.S. found that the risk for acquisition of carbapenem-resistant *A. baumannii* in a hospital-setting quadrupled with carbapenem exposure [37], indicating that carbapenem restriction in combination with infection control measures are important to reduce the prevalence of highly resistant infections caused by *Acinetobacter* spp. *Acinetobacter* spp. infections are most often acquired in hospitals in severely ill patients, and community-acquired infections are rare [33]. Community-acquired pneumonia caused by *Acinetobacter* spp. have been associated with underlying factors, such as alcoholism, smoking, chronic obstructive pulmonary disease and diabetes [33]. In our study, the majority of isolates (88.5%) came from inpatients. When comparing the proportions of MDR *Acinetobacter* spp. isolates from inpatients and outpatients, no statistically significant difference was found. When comparing non-susceptible isolates to the various antibacterial categories, no differences were found except for higher proportions of non-susceptible isolates to aminoglycosides and antipseudomonal penicillins/β-lactamase inhibitors in inpatients compared to outpatients. Other studies have reported higher rates of susceptibility in isolates derived from the outpatient settings compared to isolates derived from hospitals and nursing homes [32,38,39]. If these isolates represent true community-acquired infections, the non-susceptibility proportions found among outpatients were alarmingly high. 

### Strengths and Limitations

The data analyzed in this study are unique, since longitudinal surveillance data from India are scarce. The data were collected over a four years period and analyzed at the same laboratory. Although there could be changes in staff and laboratory practices over time, the fact that the same laboratory performed all antibacterial susceptibility testing minimizes the risk for bias due to methodological issues. A limitation of this study is that all the isolates were not tested for all the antibacterial categories. Hence, there is a possibility for both underestimation and overestimation of the proportions of MDR and non-susceptible isolates due to lack of information about antibacterial susceptibilities to all categories for all isolates. However, more than 95% of the isolates were tested for the antibacterial categories aminoglycosides, carbapenems, antipseudomonal penicillins/β-lactamase inhibitors, or cephalosporins (see legend to Figure 2). We were unable to determine whether the isolates were extensively drug-resistant (XDR) or pan-drug resistant (PDR) due to lack of information about antibacterial susceptibilities for all antibacterial categories for all isolates included in the study. Also, clinical breakpoints (inhibitory zone diameters) for polymyxins were lacking in the CLSI guideline [17]. Hence, information about antibacterial susceptibilities to all nine antibacterial categories that ECDC and CDC recommend to analyze in order to determine the prevalence of XDR and PDR isolates was not available in this study [20].

## 5. Conclusions

This study highlights the high prevalence of MDR among clinical *Acinetobacter* spp. isolates in a district in Western India. The emergence of MDR *Acinetobacter* spp. is a serious global threat to public health. Both local and national surveillance data, as well as strict infection control and antimicrobial stewardship are needed to combat infections caused by MDR *Acinetobacter* spp.

## Figures and Tables

**Table 1:** Proportions (%) of non-susceptible *Acinetobacter* spp. isolates from inpatients and outpatients according to multidrug resistance and Antibacterial category in the period from 2011–2014.

Multidrug Resistance/Antibacterial Category	Inpatients		Outpatients		*p* ^1^
Multidrug resistance	%	*n* ^2^	%	*n* ^2^	
MDR	93.6	656	91.8	85	0.522
ESBL	91.3	611	88.6	79	0.427
Antibacterial category					
Aminoglycosides	89.3	634	79.3	82	0.008
Carbapenems	86.3	649	83.1	83	0.437
Fluoroquinolones	74.6	500	70.5	78	0.444
Antipseudomonal penicillins/β-lactamase inhibitors	91.6	631	84.8	79	0.048
Cephalosporins	97.2	638	96.4	84	0.701
Folate pathway inhibitors	90.4	385	94.4	71	0.282
Penicillins/β-lactamase inhibitors	87.4	596	82.3	79	0.205
Tetracyclines	72.7	341	75.0	64	0.707

^1^ *p* < 0.050 (Chi square test, 2-tailed) indicates statistical significance of the differences in non-susceptibility rates; ^2^ *n* = total number of *Acinetobacter* spp. isolates.

**Table 2:** Proportions (%) of non-susceptible *Acinetobacter* spp. isolates from different specimen types according to multidrug resistance and antibacterial category in the period from 2011–2014.

Multidrug Resistance/Antibacterial Category	Respiratory ^1^		Other ^2^		*p* ^3^
Multidrug resistance	%	*n* ^4^	%	*n* ^4^	
MDR	96.5	375	90.1	364	<0.001
ESBL	95.5	355	86.2	333	<0.001
Antibacterial category					
Aminoglycosides	93.9	359	82.3	355	<0.001
Carbapenems	92.4	370	79.2	360	<0.001
Fluoroquinolones	78.3	285	69.9	292	0.022
Antipseudomonal penicillins/β-lactamase inhibitors	96.4	357	85.2	351	<0.001
Cephalosporins	99.2	366	94.9	354	0.001
Folate pathway inhibitors	94.1	203	88.5	252	0.038
Penicillins/β-lactamase inhibitors	91.6	346	82.0	327	<0.001
Tetracyclines	80.6	191	66.2	213	0.001

^1^ Samples from the respiratory system (bronchoalveolar lavage, sputum, throat and trachea); ^2^ Other samples (including blood and pus); ^3^ *p* < 0.050 (Chi square test, 2-tailed) indicates statistical significance of the differences in non-susceptibility rates; ^4^ *n* = total number of *Acinetobacter* spp. isolates

**Table 3:** Odds ratios (OR) with 95% confidence intervals (CI) of non-susceptible *Acinetobacter* spp. isolates for every year increase in the period from 2011–2014 from a logistic regression model.

Multidrug Resistance/Antibacterial Category	OR	95% CI
Multidrug resistance		
MDR	0.95	0.72–1.25
ESBL	1.18	0.92–1.52
Antibacterial category		
Aminoglycosides	0.97	0.78–1.20
Carbapenems	1.20	0.98–1.47
Fluoroquinolones	0.75	0.62–0.91
Antipseudomonal penicillins/β-lactamase inhibitors	1.22	0.95–1.56
Cephalosporins	0.90	0.60–1.37
Folate pathway inhibitors	1.14	0.84–1.53
Penicillins/β-lactamase inhibitors	0.97	0.78–1.20
Tetracyclines	1.03	0.83–1.28

## Appendix A

**Table A1:** Proportions (%) of non-susceptible *Acinetobacter* spp. isolates according to multidrug resistance, antibacterial category and year.

Multidrug Resistance/Antibacterial Category	2011		2012		2013		2014	
Multidrug resistance	%	*n* ^1^	%	*n* ^1^	%	*n* ^1^	%	*n* ^1^
MDR	91.1	169	95.5	201	95.9	220	89.4	151
ESBL	87.9	157	90.5	190	94.0	201	90.9	142
Antibacterial category								
Aminoglycosides	85.8	169	91.0	199	89.6	211	84.7	137
Carbapenems	81.6	168	86.1	201	87.6	218	88.3	145
Fluoroquinolones	83.3	96	83.3	126	65.3	216	72.9	140
Antipseudomonal penicillins/β-lactamase inhibitors	85.1	161	92.3	195	95.3	212	88.7	142
Cephalosporins	97.0	168	97.4	194	97.7	219	95.7	141
Folate pathway inhibitors	88.7	106	92.3	117	90.2	132	93.1	101
Penicillins/β-lactamase inhibitors	80.7	155	91.9	173	93.7	205	77.5	142
Tetracyclines	74.6	63	71.4	98	71.3	136	75.9	108

^1^ *n* = total number of *Acinetobacter* spp. isolates.

**Table A2:** Proportions (%) of *Acinetobacter* spp. isolates from inpatients and respiratory samples tested for fluoroquinolone non-susceptibility per year.

Type of Patient/Specimen Type	2011		2012		2013		2014	
	%	*n* ^1^	%	*n* ^1^	%	*n* ^1^	%	*n* ^1^
Inpatients	88.5	96	81.0	126	86.1	216	90.7	140
Respiratory samples	39.0	95 ^2^	39.7	126	61.1	216	47.1	140

^1^ *n* = total number of *Acinetobacter* spp. isolates tested for fluoroquinolone non-susceptibility; ^2^ One missing value on specimen type.

## References

[1] Clark N.M., Zhanel G.G., Lynch J.P. (2016). Emergence of antimicrobial resistance among *Acinetobacter* species: A global threat. Curr. Opin. Crit. Care.

[2] Doi Y., Murray G.L., Peleg A.Y. (2015). *Acinetobacter baumannii*: Evolution of antimicrobial resistance-treatment options. Semin. Respir. Crit. Care Med..

[3] Peleg A.Y., Seifert H., Paterson D.L. (2008). *Acinetobacter baumannii*: Emergence of a successful pathogen. Clin. Microbiol. Rev..

[4] Da Silva G.J., Domingues S. (2016). Insights on the Horizontal Gene Transfer of Carbapenemase Determinants in the Opportunistic Pathogen *Acinetobacter baumannii*. Microorganisms.

[5] Brown S., Amyes S. (2006). OXA (beta)-lactamases in *Acinetobacter:* The story so far. J. Antimicrob. Chemother..

[6] Giske C.G., Monnet D.L., Cars O., Carmeli Y. (2008). Clinical and economic impact of common multidrug-resistant gram-negative bacilli. Antimicrob. Agents Chemother..

[7] Poulikakos P., Tansarli G.S., Falagas M.E. (2014). Combination antibiotic treatment versus monotherapy for multidrug-resistant, extensively drug-resistant, and pandrug-resistant *Acinetobacter* infections: A systematic review. Eur. J. Clin. Microbiol. Infect. Dis..

[8] Cai Y., Chai D., Wang R., Liang B., Bai N. (2012). Colistin resistance of *Acinetobacter baumannii*: Clinical reports, mechanisms and antimicrobial strategies. J. Antimicrob. Chemother..

[9] Gales A.C., Jones R.N., Sader H.S. (2006). Global assessment of the antimicrobial activity of polymyxin B against 54 731 clinical isolates of Gram-negative bacilli: Report from the SENTRY antimicrobial surveillance programme (2001–2004). Clin. Microbiol. Infect..

[10] Falagas M.E., Bliziotis I.A., Siempos I.I. (2006). Attributable mortality of *Acinetobacter baumannii* infections in critically ill patients: A systematic review of matched cohort and case-control studies. Crit. Care.

[11] Playford E.G., Craig J.C., Iredell J.R. (2007). Carbapenem-resistant *Acinetobacter baumannii* in intensive care unit patients: Risk factors for acquisition, infection and their consequences. J. Hosp. Infect..

[12] Wilson S.J., Knipe C.J., Zieger M.J., Gabehart K.M., Goodman J.E., Volk H.M., Sood R. (2004). Direct costs of multidrug-resistant *Acinetobacter baumannii* in the burn unit of a public teaching hospital. Am. J. Infect. Control.

[13] Wong D., Nielsen T.B., Bonomo R.A., Pantapalangkoor P., Luna B., Spellberg B. (2017). Clinical and Pathophysiological Overview of *Acinetobacter* Infections: A Century of Challenges. Clin. Microbiol. Rev..

[14] Bac-Test Laboratory Web Page. http://www.indiamart.com/bac-test-laboratory/.

[15] Indian Census 2011. http://www.census2011.co.in/census/district/354-nashik.html.

[16] WHO WHONET Software. http://www.whonet.org/software.html.

[17] Clinical and Laboratory Standards Institute (2014). Performance Standards for Antimicrobial Susceptibility Testing.

[18] National Committee for Clinical Laboratory Standards (2015). Methods for Dilution Antimicrobial Susceptibility Tests for Bacteria That Grow Aerobically—Tenth Edition: Approved Standard M07-A6.

[19] Bauer A.W., Kirby W.M., Sherris J.C., Turck M. (1966). Antibiotic susceptibility testing by a standardized single disk method. Am. J. Clin. Pathol..

[20] Magiorakos A.P., Srinivasan A., Carey R.B., Carmeli Y., Falagas M.E., Giske C.G., Harbarth S., Hindler J.F., Kahlmeter G., Olsson-Liljequist B. (2012). Multidrug-resistant, extensively drug-resistant and pandrug-resistant bacteria: An international expert proposal for interim standard definitions for acquired resistance. Clin. Microbiol. Infect..

[21] Alagesan M., Gopalakrishnan R., Panchatcharam S.N., Dorairajan S., Mandayam Ananth T., Venkatasubramanian R. (2015). A decade of change in susceptibility patterns of Gram-negative blood culture isolates: A single center study. Germs.

[22] Gandra S., Mojica N., Klein E.Y., Ashok A., Nerurkar V., Kumari M., Ramesh U., Dey S., Vadwai V., Das B.R. (2016). Trends in antibiotic resistance among major bacterial pathogens isolated from blood cultures tested at a large private laboratory network in India, 2008–2014. Int. J. Infect. Dis..

[23] Jean S.S., Coombs G., Ling T., Balaji V., Rodrigues C., Mikamo H., Kim M.J., Rajasekaram D.G., Mendoza M., Tan T.Y. (2016). Epidemiology and antimicrobial susceptibility profiles of pathogens causing urinary tract infections in the Asia-Pacific region: Results from the Study for Monitoring Antimicrobial Resistance Trends (SMART), 2010–2013. Int. J. Antimicrob. Agents.

[24] Lob S.H., Hoban D.J., Sahm D.F., Badal R.E. (2016). Regional differences and trends in antimicrobial susceptibility of *Acinetobacter baumannii*. Int. J. Antimicrob. Agents.

[25] Mahajan G., Sheemar S., Chopra S., Kaur J., Chowdhary D., Makhija S.K. (2011). Carbapenem resistance and phenotypic detection of carbapenemases in clinical isolates of *Acinetobacter baumannii*. Indian J. Med. Sci..

[26] Abbo A., Navon-Venezia S., Hammer-Muntz O., Krichali T., Siegman-Igra Y., Carmeli Y. (2005). Multidrug-resistant *Acinetobacter baumannii*. Emerg. Infect. Dis..

[27] Agarwal S., Kakati B., Khanduri S., Gupta S. (2017). Emergence of Carbapenem Resistant Non-Fermenting Gram-Negative Bacilli Isolated in an ICU of a Tertiary Care Hospital. J. Clin. Diagn. Res..

[28] Khajuria A., Praharaj A.K., Kumar M., Grover N. (2014). Molecular Characterization of Carbapenem Resistant Isolates of *Acinetobacter baumannii* in an Intensive Care Unit of a Tertiary Care Centre at Central India. J. Clin. Diagn. Res..

[29] Sivaranjani V., Umadevi S., Srirangaraj S., Kali A., Seetha K. (2013). Multi-drug resistant *Acinetobacter* species from various clinical samples in a tertiary care hospital from South India. Australas. Med. J..

[30] Sohail M., Rashid A., Aslam B., Waseem M., Shahid M., Akram M., Khurshid M., Rasool M.H. (2016). Antimicrobial susceptibility of *Acinetobacter* clinical isolates and emerging antibiogram trends for nosocomial infection management. Rev. Soc. Bras. Med. Trop..

[31] Uwingabiye J., Frikh M., Lemnouer A., Bssaibis F., Belefquih B., Maleb A., Dahraoui S., Belyamani L., Bait A., Haimeur C. (2016). *Acinetobacter* infections prevalence and frequency of the antibiotics resistance: Comparative study of intensive care units versus other hospital units. Pan Afr. Med. J..

[32] Zilberberg M.D., Kollef M.H., Shorr A.F. (2016). Secular trends in *Acinetobacter baumannii* resistance in respiratory and blood stream specimens in the United States, 2003 to 2012: A survey study. J. Hosp. Med..

[33] Dijkshoorn L., Nemec A., Seifert H. (2007). An increasing threat in hospitals: Multidrug-resistant *Acinetobacter baumannii*. Nat. Rev. Microbiol..

[34] Chung D.R., Song J.H., Kim S.H., Thamlikitkul V., Huang S.G., Wang H., So T.M., Yasin R.M., Hsueh P.R., Carlos C.C. (2011). High prevalence of multidrug-resistant nonfermenters in hospital-acquired pneumonia in Asia. Am. J. Respir. Crit. Care Med..

[35] Sader H.S., Farrell D.J., Flamm R.K., Jones R.N. (2014). Antimicrobial susceptibility of Gram-negative organisms isolated from patients hospitalized in intensive care units in United States and European hospitals (2009–2011). Diagn. Microbiol. Infect. Dis..

[36] Goel N., Wattal C., Oberoi J.K., Raveendran R., Datta S., Prasad K.J. (2011). Trend analysis of antimicrobial consumption and development of resistance in non-fermenters in a tertiary care hospital in Delhi, India. J. Antimicrob. Chemother..

[37] Munoz-Price L.S., Rosa R., Castro J.G., Laowansiri P., Latibeaudiere R., Namias N., Tarima S. (2016). Evaluating the Impact of Antibiotic Exposures as Time-Dependent Variables on the Acquisition of Carbapenem-Resistant *Acinetobacter baumannii*. Crit. Care Med..

[38] Leung W.S., Chu C.M., Tsang K.Y., Lo F.H., Lo K.F., Ho P.L. (2006). Fulminant community-acquired *Acinetobacter baumannii* pneumonia as a distinct clinical syndrome. Chest.

[39] Oh Y.J., Song S.H., Baik S.H., Lee H.H., Han I.M., Oh D.H. (2013). A case of fulminant community-acquired *Acinetobacter baumannii* pneumonia in Korea. Korean J. Intern. Med..

